# No Need for a Cognitive Map: Decentralized Memory for Insect Navigation

**DOI:** 10.1371/journal.pcbi.1002009

**Published:** 2011-03-17

**Authors:** Holk Cruse, Rüdiger Wehner

**Affiliations:** 1 Biological Cybernetics, and Center for Excellence CITEC, University of Bielefeld, Bielefeld, Germany; 2 Brain Research Institute, University of Zürich, Zürich, Switzerland; 3 Biocenter, University of Würzburg, Würzburg, Germany; University College London, United Kingdom

## Abstract

In many animals the ability to navigate over long distances is an important prerequisite for foraging. For example, it is widely accepted that desert ants and honey bees, but also mammals, use path integration for finding the way back to their home site. It is however a matter of a long standing debate whether animals in addition are able to acquire and use so called cognitive maps. Such a ‘map’, a global spatial representation of the foraging area, is generally assumed to allow the animal to find shortcuts between two sites although the direct connection has never been travelled before. Using the artificial neural network approach, here we develop an artificial memory system which is based on path integration and various landmark guidance mechanisms (a bank of individual and independent landmark-defined memory elements). Activation of the individual memory elements depends on a separate motivation network and an, in part, asymmetrical lateral inhibition network. The information concerning the absolute position of the agent is present, but resides in a separate memory that can only be used by the path integration subsystem to control the behaviour, but cannot be used for computational purposes with other memory elements of the system. Thus, in this simulation there is no neural basis of a cognitive map. Nevertheless, an agent controlled by this network is able to accomplish various navigational tasks known from ants and bees and often discussed as being dependent on a cognitive map. For example, map-like behaviour as observed in honey bees arises as an emergent property from a decentralized system. This behaviour thus can be explained without referring to the assumption that a cognitive map, a coherent representation of foraging space, must exist. We hypothesize that the proposed network essentially resides in the mushroom bodies of the insect brain.

## Author Summary

When desert ants search for food, they often have to travel over long distances, more then ten thousand times their body lengths and then turn back to find the nest entrance. It is known from many experiments that these animals employ a skylight compass including the sun, a pedometer, and a mechanism called path integration. This means that during walking they continuously update the vector pointing from their actual position back to the nest site. In addition they use landmarks. However, based on observations of the behaviour of ants and honey bees several authors have argued that these animals finally employ a neural system that is able to represent frequently visited locations in the form of a map (a “cognitive map”). Having a map-like system available would allow the animal to find a shortcut between two separately learned locations without having learned this direct path between both locations beforehand. As such shortcuts have been observed, cognitive maps have been assumed to exist. Here we show in a simulation study based on artificial neural networks that shortcuts as observed in the experiments are also possible with a memory system using a completely decentralized architecture not including an explicit cognitive map.

## Introduction

Desert ants are extremely skillful long-distance navigators, which during their foraging journeys can leave their underground colonies for distances of more than ten thousand times their body length, and then return to their point of departure, an often inconspicuous hole in the desert ground, with amazing accuracy. Due to these feats of navigation, and the methodological ease with which the spatial layout of their outbound and inbound journeys can be recorded and experimentally manipulated, these ants have become model organisms for the study of how insects find their way in featureless as well as cluttered environments. As neurobiological and behavioural research done over the past four decades has shown (for reviews see [1]–[4]), the ant's navigational toolbox consists of a number of modules flexibly employed by the animal in a variety of ways. Among these modules is a skylight compass [5], a wind compass [6], a distance-integrating odometer [7], a path integrator combining compass and odometer information [8], [9], one and another system of landmark guidance used in place recognition and route navigation [4], [10], [11] as well as an area-concentrated systematic search routine [12]–[15].

Apart from the wealth of information now available about these various navigational systems, the question of how the insect finally combines this information to accomplish a particular task at a particular time of its foraging journey has been a matter of substantial debate. Is the information provided, e.g., by the path-integration and landmark-guidance systems combined and integrated into a ‘cognitive map’ *sensu* Tolman [16], a global spatial representation of the insect's foraging terrain, as proposed for honey bees first by Gould [17] and later more extensively by Menzel et al. [18], [19]? Or do the various navigational routines interact, simultaneously and successively, in flexible, largely context-dependent ways, with context provided by external cues and internal motivational states [1], [2], [20], [21]? As far as the neural architecture of the insect's navigational toolkit is concerned, the former hypothesis implies that the domain-specific processing modules feed their information into a ‘central integrator state’ [22], while the latter hypothesis proposes that the domain-specific modules are interlinked within a distributed system [23].

The debate is not simplified by the fact that Tolman has defined the term ‘cognitive map’ in an only loosely way: “… the incoming impulses are usually worked over and elaborated in the central control room into a tentative, cognitive-like map of the environment. And it is this tentative map, indicating routes and paths and environmental relationships, which finally determines what responses, if any, the animal will finally release.” (Tolman, 1948 - p. 192). His use of the terms ‘cognitive map’ and ‘central control room’ is reminiscent of what cognitive scientists nowadays often call ‘global neural workspace’ (see [24] for a review). Functionally, this term describes the idea that different elements stored separately in memory can dynamically be connected, for example to allow for the invention of new behaviours. Applied to the navigation problem, this means that different memory elements, for example vectors representing the locations of two food sites, may be used for common computation. In contrast, in a decentralized or, as it is often called, reactive system such a combination of separately stored vectors for computation is not possible. In such a reactive system a memory content can only be used within the context in which it has been learned. A basic functional difference between both types of systems is that the cognitive system allows for high flexibility but is slow because the search for new combinations of memory elements requires time, whereas the reactive architecture allows for fast, though inflexible reactions. (For an example how a reactive system can be transformed to constitute a cognitive system see [25]).

In the present account we follow the latter idea and design an architecture that allows us to test whether a distributed network based on the main experimental results obtained in the study of desert ant navigation is able to simulate the behavioural performances of ants and bees – and especially those performances that have not been used in designing the architecture. If such a solution was definitely found, a cognitive interpretation could be given up as it represents a more complex hypothesis. If no such solution was found, the probability for the existence of a cognitive map would increase.

The basic experimental results on which the simulation is based are the following (for references see the papers cited above and the references therein):

1. Path integration. There are various ways in which a path integration (PI) system could work – egocentrically or geocentrically, continuously or discontinuously, based completely on idiothetic cues or employing external cues as well - but for our present purposes it is not important to differentiate between these possibilities. We just assume that the animal possesses a PI vector memory, in which the nest-to-food vector, and reversed in sign the food-to-nest vector, is stored (‘reference vector’), and that at any one time during an inbound and outbound trip the animal compares the state of its ‘current vector’ with the reference vector. If the former matches the latter, the path integrator has acquired its zero-state, and the animal has reached the goal. Then the current vector is reset to zero, but the reference vector remains in memory. For the sake of illustration let us follow an ant that leaves its home and sets out for a foraging journey. While the ant is on the way, its PI system computes and continually updates a current vector. When a food item has been found, this current vector is stored and becomes a reference vector. When the ant decides to visit the same food source at a later time again, its current vector (which is zero when the ant leaves the nest and increases in length as the ant proceeds on its way) is continually subtracted from the reference vector. Once current and reference vectors coincide, the ant has reached the food site. Upon departure from that site the reference vector is reversed in sign, and the PI system starts to work again in the way described above.
2. Area-concentrated search. As any PI system is prone to cumulative errors, a ‘zero-vector ant’ will not have arrived exactly at the goal, but at some location close to it. The ant then starts to perform systematic search movements that are centred about the point at which the PI had reached its zero state. It is important to note that during the entire search the path integrator keeps running, and that it is reset to zero only after the ant has entered the nest.
3. Landmark guidance: Place learning. To further aid the localization of the (usually inconspicuous) nest entrance, the ants make intensive use of landmark information. Experimental results indicate that one or several ‘snapshots’ of the landmark scene at the home site are taken and memorized. Later this snapshot view, or an individual signpost within this view, is used to guide the animal from any place near the home site to the nest entrance. The underlying mechanism is best described by an attempt to match the stored snapshot with the currently seen view. This matching mechanism provides a direction defined relative to the landmark. We will call this landmark a ‘home landmark’. Correspondingly, landmark views can be acquired at the food source (‘food landmarks’).
4. Landmark guidance: Route learning. In addition to landmarks at the nest and food sites, landmarks distributed in the area between these two sites may be used for navigation. Any such landmark view can be associated with a specific walking direction termed ‘local vector’ (although it still remains to be fully established to what extent the length of this ‘vector’ is specified). As these landmarks are visited by the animals *en route*, they will be called ‘route landmarks’.

In both types of landmark - place (nest and food) landmarks and route landmarks – long term memories enable the animal to compare the actual visual inputs with the memory stores. A best-match procedure decides whether one of the stored memories is activated, a walking angle is computed and delivered to the output stages. The difference in the use of both types of landmark is that in the case of the place landmark (or landmark scene) the input is continuously compared with the stored view, whereas in the case of the route landmark, once seen, a walking direction is determined, and the landmark is then no longer attended. Hence, in the case of a place landmark the walk leads to an even better match, while in the case of a route landmark the walk leads the animal away from the landmark. Furthermore, recognition of a landmark suppresses the influence of the local vector provided by an earlier landmark.

1. Multiple memories. Ants are able to learn and store (i) more than one reference vector pointing to more than one food site and (ii) several landmark-defined positions within their foraging terrain, and (iii) more than one landmark-defined route.
2. Hierarchical relationships. If a familiar landmark is recognized, ants follow the corresponding (landmark-associated) local vector rather than their PI vector.
3. Motivational states. Landmarks are stored and retrieved only in specific contexts. One of the most important (internal, motivational) contexts is whether the ant is on its ‘outbound’ or ‘inbound’ trip, i.e., whether it walks from home to food or from food to home, respectively.

## Model

### Structure of network

**Figure 1:**
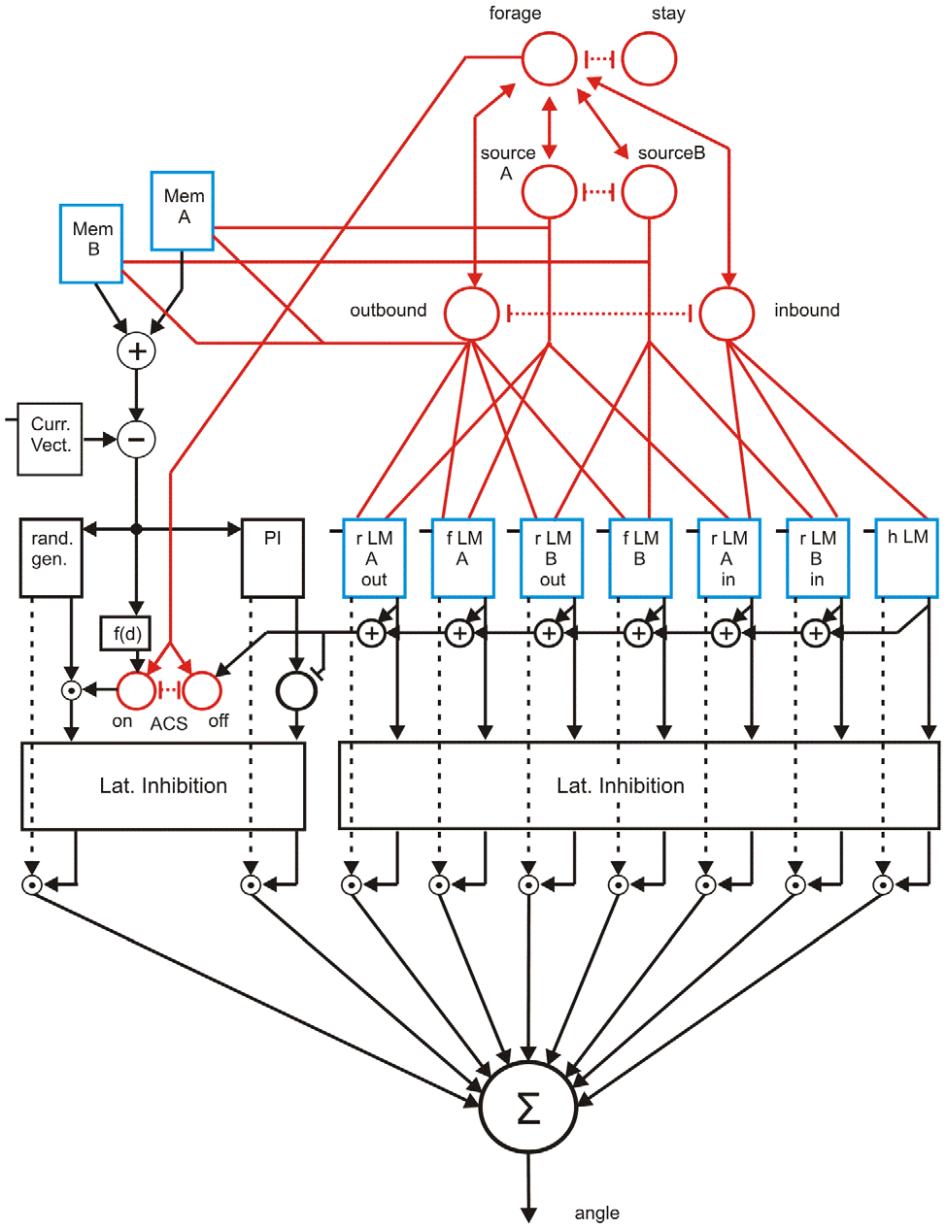
The network controlling path integration and landmark navigation. Eight motivation units (red), a bank of memory elements shown in blue, (Mem A, Mem B) and seven further elements (two food landmark elements, fLMA, fLMB, one home landmark element, hLM, and four combined route landmark elements, rLMAout, rLMBout, rLMAin, rLMBin, lower right). The path integrator is schematically depicted at lower left (Curr. Vect, PI). Box rand.gen. and the motivation units ACS on, off control “area-concentrated search” walks. For further details see text.

**Figure 2:**
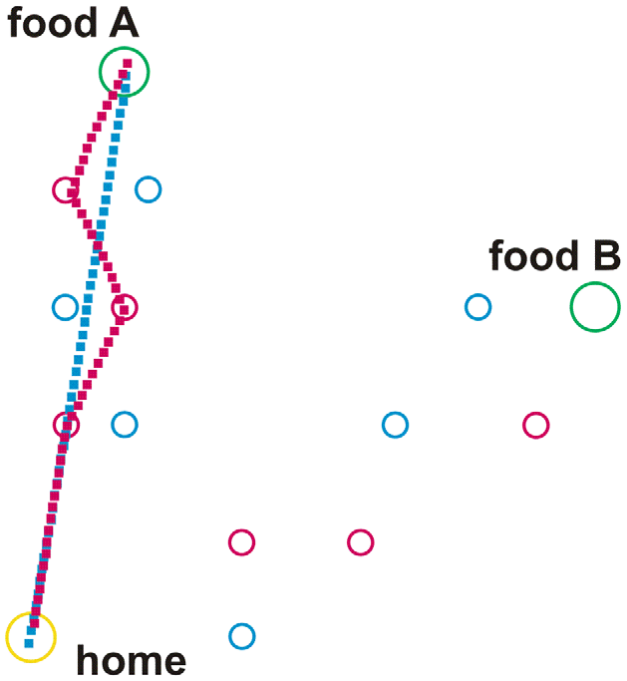
Walk from home to food source A and back. The catchment areas of the landmark elements home, and the two food sources A and B are shown by circles coloured in yellow and green, respectively. Catchment areas of route landmarks are shown in red or blue for outbound and inbound landmarks, respectively. Outbound walk is depicted by red squares, inbound walk by blue squares. For further explanations see text.

**Figure 3 pcbi-1002009-g003:**
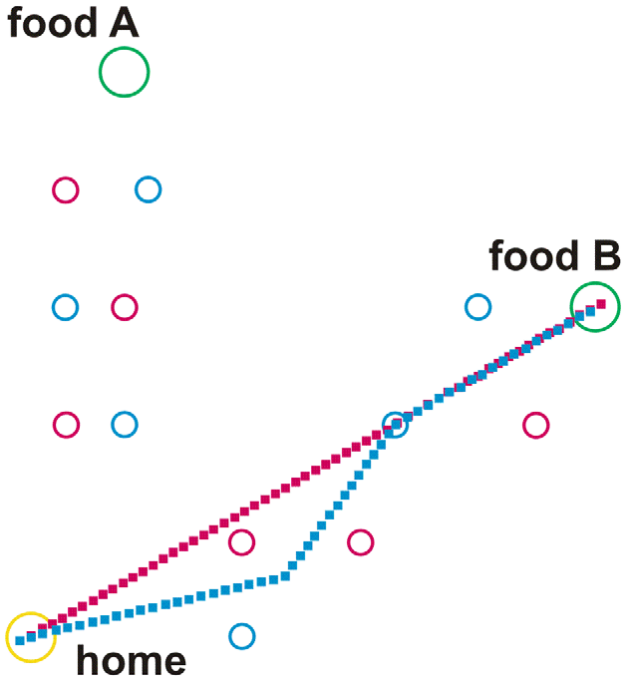
Walk from home to food source B and back. The catchment areas of the landmark elements home, and the two food sources A and B are shown by circles coloured in yellow and green, respectively. Catchment areas of route landmarks are shown in red or blue for outbound and inbound landmarks, respectively. Outbound walk is depicted by red squares, inbound walk by blue squares. For further explanations see text.

**Figure 4 pcbi-1002009-g004:**
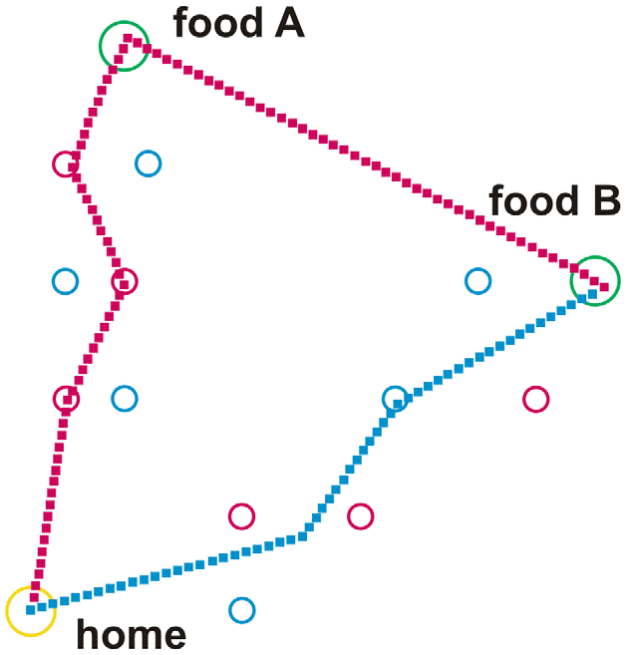
Walk from home to food source. A. As there is no food, the agent performs a short cut to food source B. After having found food, it returns to home using one route landmark. As the local vector to the next route landmark is too short, path integration takes over. The catchment areas of the landmark elements home, and the two food sources A and B are shown by circles coloured in yellow and green, respectively. Catchment areas of route landmarks are shown in red or blue for outbound and inbound landmarks, respectively. Outbound walk is depicted by red squares, inbound walk by blue squares. For further explanations see text.

**Figure 5:**
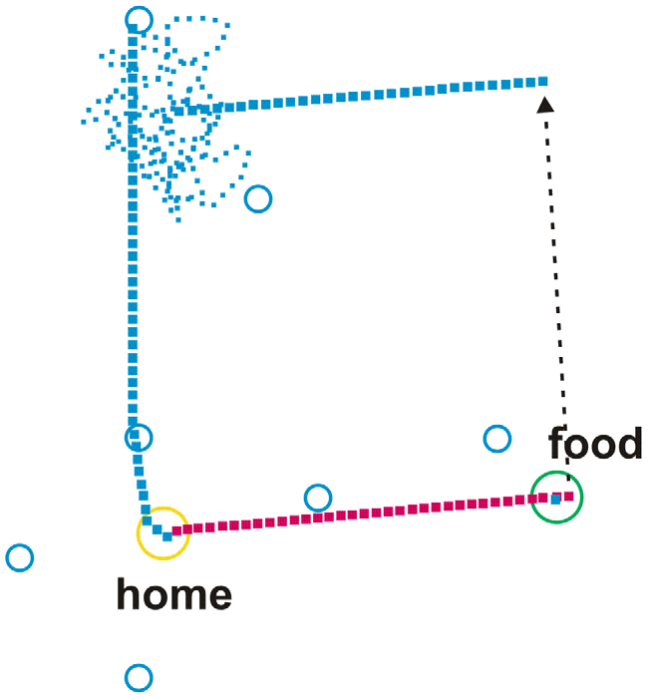
Flight path of a honey bee that is transported from the food source to a place about north of the feeder (dashed black arrow). After being released, the simulated bee performs a straight flight following the path integrator, then performs a search flight (small dots) until the bee by chance meets a known route landmark. The catchment areas of the landmark elements home and the food source are shown by circles coloured in yellow and green, respectively. Catchment areas of route landmarks are shown in blue for inbound landmarks. Outbound trip is depicted by red squares, inbound trip by blue squares. For further explanations see text.

To simulate basic properties of ant navigation during foraging the following network has been implemented (Figure 1). The net consists of three main parts: (i) a system being responsible for path integration (PI, eventually also termed vector navigation, [26], [27]) as depicted at the left hand side of Figure 1, (ii) a recurrent network controlling different motivations (Figure 1, upper right, in red), and (iii) a bank of procedural memories (horizontal row of blue boxes in the center of Figure 1). Each of these procedural elements receives sensory input, indicated by the short bar at the upper left of each box (e.g., visual input concerning a specific landmark), and input from the motivation network. As outputs these memory elements provide vectors determining a walking direction (relative to an absolute external reference system defined by a compass). The path integrator system represents a procedure, too, containing memory elements (Figure 1, blue boxes, upper left) and providing a corresponding output vector. All these sensorimotor, or procedural, memories are independent of each other. Their output values undergo a weighted summation. The weights are dynamically determined by a lateral inhibition network that, based on the vector lengths, determines a confidence or salience value for each memory element. To keep the simulation as simple as possible, learning processes as such are not simulated, but memories may be switched off or on by hand to simulate different learning states. To study the behaviour of an agent controlled by this network we apply an environment containing a nest (home) and two food sources (A and B). In the experiments presented in Figures 2, 3 and 4, there are 12 route landmarks distributed over the space between home and the two food sources (Different landmarks are used in a later experiment, see Figure 5).

### Main procedures

In the following the different parts of the network will be described in more detail. Let us begin with the *path integrator*. As detailed models are available (e.g., [28], [29], for a comprehensive review of types of model, see [30]), this part is simulated here in an abstracted form only. The path integrator (not shown in Figure 1) provides the ‘current vector’ (Figure 1, left, Curr. Vect.). If a food source is detected, the actual current vector is stored as a long term memory (e.g. Mem A, Figure 1, upper left) and is termed ‘reference vector’. The reference vector might, in the simplest case, only be stored as a kind of short term memory that is cleared after the nest has been reached again. If, however, the food source is rich enough, so that further visits are intended, this vector might (and can be, by bees and ants) stored as a long term memory element. Therefore, the quality of the food appears to be a crucial motivational factor influencing learning.

The stored reference vector pointing from the home position to that of the food source can be used to later control visits of this food source by subtracting the current vector from the reference vector (Figure 1, upper left, circle containing a subtraction symbol: Mem() – Curr. Vect.). The difference provides angles describing the walking direction (defined relative to an absolute direction given by a compass) and the remaining distance to the goal. The same system controlling these ‘outbound’ walks can be used to control the walks from food back to home (‘inbound’ walks), when the goal vector refers to the home site, i.e., has zero length. The output of this system is represented in Figure 1 by box PI. Before further computation, the output vector of this box is normalized to show a length of 1.

#### Motivation network

To use this system for navigation, at least one basic decision must be made. Is the agent in inbound mode or in outbound mode? A further decision is necessary if the agent has learned the position of two different food sources, A and B. Which food source should be selected? On a higher level, the agent may furthermore have the ability to choose between foraging behaviour and any other type of behaviour. To take a simple example, we use as a second behaviour ‘stay’, i.e. stay in the nest. All these decisions are formed by a recurrent neural network (Figure 1, red; units connected by double headed arrows or inhibitory connections), the units of which are called ‘motivation’ units here. A motivation unit can adopt a value between 0 and 1. The connections between these units (for technical details see below) are designed in a way that the three pairs (inbound – outbound), (forage – stay) and (sourceA – sourceB) are connected by mutually inhibitory weights (Figure 1, dashed red lines). Such a connection has the effect that only one unit of each pair can be active after the net has relaxed to a stable state (or attractor). If unit ‘stay’ is active, unit ‘forage’ is inactive and as a consequence all other units positively connected to unit ‘forage’ are inactive, too. If unit forage is active, one of the units sourceA or sourceB is active, while the other one is inactive. The output of these units determines which memory content can be used by the vector navigation system (see input to boxes MemA and MemB in Figure 1). The decision between units sourceA and sourceB may be a random decision or may be determined by other contents of the agent's memory, e.g. the quality of the food, values being given as input to the motivation units (not shown in Figure 1). Correspondingly, a decision between inbound and outbound is made by sensory input to the corresponding units. For example, if the agent leaves home, activation of the unit outbound is stimulated. If food has been found, this stimulus activates unit ‘inbound’ (not depicted in Figure 1, either). The motivation unit for ‘outbound’ controls the output of the memory elements (MemA, MemB). If outbound is switched off, their output will be zero. Thus, path integration depends on two motivations, sourceA or sourceB, and inbound or outbound, as is the case for the procedural memories that will be described next.

#### Route landmarks

After having learned the global vectors (MemA, MemB) pointing to the corresponding food sources, the agent may in addition learn specific route landmarks situated anywhere in the landscape between home and food sites. As mentioned in the Introduction, ants can learn ‘local vectors’ associated with each landmark or sets of landmarks. When having perceived a learned landmark, the animal follows a specific angle (relative to an absolute direction given by a compass). There is no strong experimental evidence how exactly ants and bees detect the walking direction associated with a route landmark, but there is strong evidence that they do [11], [31]. In the simulation the walking direction is simply provided as soon as the agent enters the catchment area. It is also not fully clear yet from behavioural experiments whether or to what extent the length of this ‘vector’ is specified [32], [33]. As it has been found that ants indeed use landmarks for navigation, the simplest assumption is that the ant follows the local direction until it finds another known landmark, which then specifies a new angle. However, there might be a maximum length to be followed.

For the simulations shown in Figures 2, 3 and 4 we assume that the agent has learned three *route landmarks* walking from home to food source A and three other route landmarks for the way back from A to home. Similarly, it has learned three further route landmarks on the way from home to source B (outbound) and three more route landmarks from B to home (inbound). The three long term memory elements belonging to one trip are graphically packed into one box. As we have four trips, four such boxes are required (see Figure 1, rLM Aout, rLM Ain, rLM Bout, rLM Bin). To simplify the drawing, only one output vector is depicted. Actually, however, there are three such vectors, one for each landmark, as the three elements of each box are specified independently. It is important to emphasize that no information is stored that concerns the spatial/temporal order according to which the landmarks may be visited.

A given landmark memory is only active if (i) the appropriate motivation unit (inbound or outbound) and (ii) the motivation unit concerning the actual goal (sourceA, sourceB) are active. In other words, the procedure driven by a particular landmark is only allowed to be in charge if both necessary motivations are activated. Therefore, as for the path integration memories, each box in Figure 1 is shown to receive two motivation inputs. Reaction to such a landmark is simply simulated as follows. As soon as the agent enters the ‘catchment area’ of this landmark (indicated by circles in Figures 2 to [Fig pcbi-1002009-g003] [Fig pcbi-1002009-g004] 5), the corresponding angle value is generated provided that activation by both appropriate motivations have reached threshold.

How to deal with the output values provided by the procedural memories? Each procedure provides an output vector, consisting of an angle (Figure 1, dashed arrows) and a vector length (Figure 1, solid arrows), see Computational Details in Text S1 for details of implementation. The vector of a route landmark vector is set to a length of 1 when the landmark was perceived. However, when the walk continues, some kind of ‘forgetting’ takes place such that the vector length decreases stepwise by 0.05 for ten steps, then stays constant at 0.5 for another five steps. After a given number of steps, in our case 15 steps, the vector length of this route landmark decreases to zero. This decrease, the detailed time course of which is not critical, has the effect that a newly recognized landmark can override the memory of the landmark detected earlier. Recall that vector length does not control movement directly, but is only used as salience value, the usage of which will be explained below (Sect. Cooperation among Procedures).

#### Home and food landmarks

As mentioned in the Introduction, ants have been shown not only to use route landmarks that are freely distributed in the landscape between the home and the food site, but most importantly also landmarks that define particular places such as the home and one or another frequently visited food site (referred to as home landmark and food landmark, respectively). In our simulation we assume that there are three such procedures stored, that when stimulated provide an angle leading the agent to the corresponding goal, i.e., to either food source A, food source B, or home (Figure 1: fLMA, fLMB and hLM, respectively). These landmark elements consist of three signposts each. The signposts are, however, not stored as separate elements, as is the case for the route landmarks explained above, but form one landmark, i.e., are stored in the form of a ‘snapshot’. Again, there is a number of simulation approaches available in the literature. In our case, each home- or food-landmark memory element is represented by a simple algorithm [34], which determines the mean value of three vectors calculated from learned vectors and the vectors pointing from the agent's position to the position of the landmark. Length of the mean vector is set to a value of 1. As for the landmark vectors explained above, the food memories and home memories are controlled by double motivational input (as depicted in Figure 1). (The salience value, i.e., the length of the vector, might be smaller than 1, representing sensory input being deteriorated by noise, but this case has not been simulated here).

#### Area concentrated search

Before we continue to explain how the output values of all the procedural memories are combined to determine a walking direction, let us describe the procedural element that is responsible for the final *area concentrated search* behaviour. As mentioned in the Introduction, a so called zero-vector ant, i.e., an ant that has arrived at the goal but has not succeeded in pinpointing it exactly, engages in a specific type of search walk, the so called area concentrated search (ACS). In our simulation such a zero-vector ant corresponds to an agent whose input to the PI element is zero. Instead of explicitly implementing a specific area-concentrated search procedure, we simplified the system in the following way. Box (rand.gen.) depicted in Figure 1 represents a simple random process that changes the actual walking direction by a randomly chosen angle. In our simulation, the actual angle change could be selected from an equal distribution out of an interval between 0.7 and −0.3 [rad], thus providing a bias that supports a tendency to counterclockwise turns. Search walk is switched on if, during inbound walks, the current vector is about zero indicated by the input from Curr. Vect. in Figure 1. The length of the output vector of box (rand. gen.) is 1. However, this vector length is changed depending on the length of the current vector and on time elapsed since the search walk has started in the following way. Vector length is 1 for current vector lengths smaller than 3.3 length units, then linearly decreases to zero until the length of the current vector has reached a value of 20 units, thus roughly approximating a Gaussian function. As ants increase their searching area when the duration of the search is increasing [12], in the simulation these two figures linearly increase with searching time until the maximum values of 33 and 200 have been reached, respectively. This function is represented by box f(d) in Figure 1. (We did not implement the property of ants that the search area is also increased when the preceding travel was longer [14], [15]). The resulting vector length activates a specific motivation unit, ACSon, which determines the actual length of the output vector of the random generator (Figure 1, multiplication of rand. gen. output and ASCon output). The motivation unit ACSon is coupled, via mutual inhibition, to a second motivation unit, ACSoff. Both motivation units receive excitatory input from the motivation unit ‘forage’. As the search walk should be switched off as soon as a relevant landmark is observed, vector lengths of all landmark elements are summed and this value activates the ACSoff unit, which in turn inhibits the unit ACSon. As due to experimental results the output of the path integrator is not applied when a landmark procedure is active, this sum is also used to zero the vector length of the path integrator output. Before further treatment, vector lengths of the search procedure and of the path integrator are multiplied by a factor of 0.2 (not shown in Figure 1). This has been done to guarantee that in the final step, as explained next, signals from landmarks can override signals from the ACS procedure and the PI procedure.

### Cooperation among the procedures

Finally we must implement ways of how *to combine the outputs of the different procedures*: To accomplish this task the angular output values of the active procedural memories are subject to a weighted summation. The weights, or ‘salience values’, are determined in the following way. The vector lengths (but not the angle values) provided by the procedures are given to a one-layered feedforward lateral inhibition network. This network is shown by the boxes “Lat. Inhibition” in Figure 1. Lateral inhibition has the effect that there will be one winner while all other output values are zero or nearly zero when there is a large difference between the competitors. If the difference between two strong competitors is small, there will be no winner but only some kind of minor decrease of both salience values. However, the weights of the Lateral Inhibition network itself are chosen sufficiently strong to guarantee that in the simulation, with one exception noted below, there will always be one winner. In our Lateral Inhibition network, the connections between the units are basically symmetric using fixed inhibitory weights of b = −0.4. There are however two exceptions from complete symmetry. First, within the landmark procedures, there are inhibitory influences from the food- and home elements to those representing the route landmarks, but there are no influences in the opposite direction. This structure has the effect that information gained from landmarks signalling food or home will be weighted stronger than information signalling route landmarks. Second, the connections between the path integrator and the random walk procedure are separated from those of the landmark procedures (indicated by the separation of the two boxes “Lat. Inhibition” in Figure 1). In the former case, there are situations were both vector lengths may show similar values leading to a mixed contribution of the random generator output and the path integrator output.

How are the salience values used that are produced by the Lateral Inhibition network? As indicated in Figure 1, each procedure has two output values, the vector length (full arrows), and the angle, represented as a vector with normalized length (dashed arrows). The vector is now multiplied by the salience value and then all these vectors are simply summed up (depicted by the unit marked by Σ in Figure 1). Therefore, the salience values weight the contribution of the different procedures. Summing up the weighted vectors and finally using the angle of the resulting vector would in principle correspond to calculation of the weighted mean of all angles. However, as mentioned above, there is always only one procedure exhibiting a salience value much higher than those of the other procedures. In particular, the salience of the PI element is zero whenever any landmark memory is active. This is guaranteed by the inhibitory influence mentioned above in the context of the area concentrated search. As explained earlier the network only provides an angle determining the walking direction. The length of the motor output vector, corresponding to velocity, is not controlled by the network but assumed to be a fixed value of ten length units in the simulation.

An interesting case occurs in a zero-vector ant. In this state the salience of the random generator is relatively high if no landmark is perceived. Therefore random selection of angles governs the output. However, as during these random movements the current vector increases again, there will be a blending of the outputs of both procedures and, if the current vector is long enough, path integration may control the output completely. As a result, an area-concentrated search can be observed even if a corresponding procedure is not explicitly implemented. As mentioned above, if a landmark is perceived, the latter overrides the salience value of the path integrator and the random procedure, due to the influence of the summed output of the landmark procedures (see Figure 1, horizontal black arrows below the blue boxes and above the Lat. Inhibition network).

For further information see Computational Details in Text S1 and [35]–[40].

## Results

Using this network, several experiments have been simulated using the environment depicted in Figures 2, 3 and 4. In the first type of experiment, all route landmark memories are switched off by hand, which corresponds to the situation that route landmarks have not been learned yet. Depending on which motivation unit - sourceA or sourceB - is stimulated stronger, the agent starts from home to the corresponding food source and then returns to the home site, all based on path integration.

The same happens in the second type of experiment in which route landmarks memories are activated, but in which the agent does not happen to touch the catchment area of any landmark. Then the agent again moves in a straight line from the home to the food site. However, if a catchment area is entered, the agent is controlled by the local vector of this landmark. An example is shown in Figure 2. The agent starts from home (yellow circle) to food source A. Outbound moves are depicted by red squares, inbound route landmarks by blue squares. The agent is first controlled by path integration and thus follows a straight line which points from home to site A. As the agent reaches the catchment area of the first route landmark (red circle), the corresponding memory element takes over control. We have chosen the direction and the length of the local vectors in such a way that the agent will normally meet another landmark. The second landmark will then guide the agent to the third one. The last landmark provides an angle leading to the food source. In this final section, the network provides redundant information as both the local vector and the PI vector point in the same direction. On the way back (inbound routes and inbound route landmarks are depicted blue), the agent does not meet the catchment area of any inbound route landmark, so that it is completely controlled by its path integrator. Accidentally, as depicted in this example, the agent meets an outbound (red) landmark. However, this landmark stimulus does not influence the agent's behaviour at all, as the outbound motivation is zero. If the agent's position had been changed by the experimenter in such a way that the agent would have met one of the inbound route landmarks (depicted in blue), the agent would have been controlled immediately by landmark navigation (not shown in Figure 2 and 3, but see Figure 5).

Figure 3 provides an example in which the outbound walk is governed by path integration. On the way back the agent meets the catchment area of a route landmark (inbound, depicted in blue). Therefore the agent is now heading towards the (blue) landmark positioned near the lower margin of the environment. However, the local vector is not long enough. Notice that when the local vector has been run off before the next landmark is met, as is the case here, the path integrator again governs the agent's behaviour and leads the agent directly to the goal, in this case to the home site.

Next we describe a situation in which the ant has learned to visit two food sites (food A and food B) by running from the nest site independently to either food A or food B. Now let us assume that it has once arrived at site A but does not find food there (because the reward has been removed by the experimenter). Would it then run directly to site B? As illustrated in Figure 4, the agent leaves home walking to food source A, because motivation unit sourceA is highly activated. However, as the agent having arrived at site A is unsuccessful there, the value of the motivation unit sourceA is decreased (the input for stimulus ‘food’ is not shown in Figure 1). Due to the properties of the motivation net – the motivation unit ‘forage’ is still running – motivation unit sourceB is activated. Consequently, the agent is immediately heading towards food source B (Figure 4). This section of its path is controlled by path integration, as the agent does not meet any landmark. (If the agent had met an outbound route landmark associated with food source B, this landmark element would have guided the agent to site B). After the agent has arrived at food site B and has been rewarded there, the inbound motivation unit gets activated and the outbound motivation unit shut off. The agent would now move back towards home – exactly as a real ant does ([2] and B. Voegeli, M. Knaden and R. Wehner, unpublished results).

To avoid possible misunderstandings let us recall that except for the current vector within the PI system, our network does not have the ability to subtract two vectors stored in any of the memory elements (for example two food vectors). If this were possible, the behavior of the agent could be explained as resulting from a subtraction (B-A), because the resulting vector describes the route taken by the agent. Actually, however, only the memory of vector B is used. This is possible because the effect produced by vector A is given in the form of the already performed movement from the nest to site A. With its current vector numerically corresponding to vector A, the agent now continues to walk until its current vector state equals that of its reference vector, i.e. vector B, and when this has been accomplished has arrived at site B. Steering along the novel route A-to-B has been governed completely by the ant's path integrator. Once food has been picked up at site B, the ant reverses the sign of its reference vector, which in our model is realized by switching off the output of memory element B, and walks directly home to the starting point.

To what extent can this approach be applied to the map-like navigation behaviour described in honeybees? Menzel and co-workers [19] recorded the bees' round-trip flight paths by applying the harmonic radar technique as introduced for such purposes by [41]. First the bees were allowed to acquire landmark information about the surroundings of the hive for about three to six days. Then they were trained to a food source, say, east of the hive. After they had filled their crops, they were captured and transported to another place, e.g., to the north of the feeder. Upon release they first performed a straight flight path pointing westwards, and hence obviously controlled by the state of their current path integration vector. After they had flown off this home vector, i.e., after their path integrator had reached its zero state, they started search flights consisting of ever widening loops. At one or another point, dubbed ‘homing point’ by the authors, most of the bees started a direct flight back towards the hive. Menzel et al. [19] argue that at these homing points the bees had read their map, determined the relative positions of the homing point and the hive, and had then been able to compute their homeward course appropriately. Some bees flew back in the direction of the feeder (i.e. south-east in our example) rather than to the hive, to which they returned only after having passed the feeder.

The bees' behaviour described above can be explained by our model as well. We assume (see also [23]) that at the ‘homing points’ the bees rather than having resorted to a coherent map-like representation of their foraging space had associated familiar landmarks with local vectors pointing - may be not directly but approximately - in the home direction. To simulate this behaviour, we use another environment. Our artificial landscape now contains a home site, one food site and seven inbound route landmarks. Figure 5 shows an example. The simulated bee uses path integration for searching the food site (red squares). After having been transported from the food site to a release site to the north (indicated by a black arrow), the simulated bee performs a straight path controlled by path integration and then starts its search flights (for details see Supporting Information in Figures S1, S2 and Results Concerning Area Concentrated Search in Text S2). As soon as it meets a known landmark during these search flights, it directly moves home by employing the learned local vectors associated with that landmark. The local vector may point either directly to the home site or to another landmark.

A behavior less easily to be explained is provided by those bees that at the ‘homing point’ headed first to the feeder. In our model we assume that during their search flights these bees as well as the majority of tested bees have encountered a known inbound landmark pointing to the home site. We further assume (in accord with Menzel et al. [19]) that both the inbound unit and the outbound unit are activated. Under the assumption, that the mutual inhibitory connections are due to habituation, the activation of both units will start to oscillate [42]. In other words, both outbound and inbound motivation units are active in an alternating fashion. Thus, after the agent has performed the straight path controlled by the path integrator, and then, during a search flight, has found an inbound landmark, the inbound unit allows for activation of the corresponding inbound landmark element (as is assumed for the other bees, too). The outbound unit, on the other hand, triggers the path integrator, if there are no outbound landmarks around in this part of the environment as it is the case in our example. As a result, the bee will come across the site of the food source again. - As an alternative interpretation (which has been provided by one of our anonymous reviewers), the bee might have found an outbound landmark which then would have led it directly to the food source. - When it perceives that site, which it had actually – and successfully – visited before, the motivation to approach the food source again (the activation of the outbound motivation unit) might be decreased, so that the bee's behaviour would now be dominated by the active state of the inbound motivation unit. Hence, our fully decentralized model system can account even for the behaviour of the feeder-directed bees. What of course remains to be answered is the question why in these bees the outbound motivation state is activated at all, i.e., alongside the inbound state.

## Discussion

What have we gained? Let us try to answer this question by summarizing the elements and functions of the network presented in this account. What we propose is an artificial neural system that consists of a network allowing for both path integration and landmark guidance.

 Each memory element receives a direct sensory input, which might have already been pre-processed in certain ways. In the case of the path integration procedure, there is some pre-processing necessary, which comprises a subtraction of the current vector from the reference vector stored in long term memory. The output values provide an angle value and a vector length and are controlled by input from motivational units. The vector length values are subject to a competition based on lateral inhibition. The vector length values are not responsible for the determination of the forward walking component (i.e. the velocity), but are only used for determining the salience values. The resulting salience values are used to weight the angle values proposed by the different procedures. These weighted angle values are simply summed up to provide the resulting walking direction. The complete network contains three types of connectivities that support the decision on which procedure may contribute to the final output. (i) The motivation net switches on or off groups of procedural nets. Only procedures that belong to both a given food source and a given state (outbound or inbound) may possibly be activated at the same time. (ii) As, however, the landmarks of an activated group are spatially separated, two landmarks of this group could only be candidates of simultaneous activation if they follow each other in a short enough distance so that the memory for the first one has not yet been completely erased when the second landmark is occurring. In this case the decision is forced by the Lateral Inhibition net. Weights of the Lateral Inhibition network are chosen in a way that in general there is one procedure winning the competition. (iii) The third type of influence suppresses the output of the path integrator and the ACS procedure as long as any landmark procedure is active (see Figure 1, horizontal black arrows below the blue boxes and above the Lat. Inhibition network). The information concerning the absolute position of the agent is present, but resides in a separate memory element (Figure 1, Current Vector) that can be used to control the behaviour, but cannot be used for computational purposes within the system outside the path integrator subsystem. Apart from the Current Vector, subtraction between any of the vectors stored in the memory elements is not possible. Activation of the motivation unit “forage” is not only required within the motivation network, but is also used to drive forward walking (not shown in Figure 1). These two output signals, forward walking and angle, can directly be applied to a network controlling six-legged walking, as for example Walknet [43].

It is important to note that there is no information exchange between the different memory elements. Each memory element only has access to its “private” data. In other words, there is no cognitive map implemented in the sense defined in the Introduction. Nevertheless, the system allows for novel shortcuts, a behavioural property that is often used as a strong hint for the existence of a cognitive map. This result shows that there is no need to explicitly implement a procedure that is responsible for controlling shortcuts. Furthermore, no explicit procedure is necessary to produce an area-concentrated search. In the simulation, the latter behaviour results from a blending of the output of a random generator and the path integrator.

One might be inclined to argue that the reduction of directional outputs combining numerous decentralized navigation modules to a single output is conceptually related to the “central control room” of Tolman. However, the final summation of all outputs simply reflects the fact that there is one motor output system onto which all memory elements necessarily have to be projected. It appears not to be sensible to equate this simple projection onto a common motor output, formally a summation, with an operation corresponding to Tolman's central control room in which “the incoming impulses are worked over and elaborated [ … ] [to] finally determine[s] what responses, if any, the animal will finally release”. The introduction of a specific term like cognitive map to simply characterize the property that there is one common motor output is definitely not justified. Rather, as has been mentioned in the Introduction, we propose to define the term cognitive map along a borderline that characterizes a qualitative step between the continuum of different types of reactive systems and a ‘cognitive’ system in the following sense. A cognitive system - and therefore the application of a cognitive map - allows for exploitation of memory elements independently of the context in which these elements have been acquired (see [25] for an example).

Is it possible to experimentally distinguish between our hypothesis and that of a cognitive map? Assume that the experiment shown above (Figure 3) is performed such that, after having learned the locations of food source A and food source B, the animal is not allowed to walk from then nest to source A, but is cought when leaving the nest and then released – still as a zero-vector ant (see above) - inside the catchment area of food source A. If there is no food at source A, the agent applying our network will now activate the motivation unit of source B and then follow a path not characterized by vector (B-A), but by vector B. This means that it will normally not move towards source B. In contrast, an agent equipped with a cognitive map in the sense defined above would be able to perform the vector subtraction (B-A) and therefore steer the shortcut route.

Taken together, our network is able to “allow[s] the bee to perform novel shortcuts and to choose between two potential goals, the hive and the feeder” ([19], p. 3044). However, contrary to the authors' conclusion, the spatial behaviour observed in the bees does not necessarily mean that a ‘cognitive map’ is in charge, which Menzel and his coworkers define as a system in which “spatial relations between environmental features [are] coherently represented” ([19], p. 3045) - as “a common spatial memory of geometric organisation (a map) … as in other animals and humans” ([44], p. 429).

Where in the brain might the presumed structures defined by our network model be located? Neuropils that immediately spring to mind in the bee's and ant's ‘forebrain’ are the mushroom bodies (corpora pedunculata). With their massively parallel processing lines, their “numerous subunits each served by its own arrangement of inputs and providing its own outputs” ([45], p. 281) as well as their recurrent network connections between other protocerebral neuropils and the mushroom body lobes they are likely candidates for housing our network structures. The longitudinal subdivision of the lobes indeed suggests longitudinal multiplexing of discrete integrative networks [46] that form an essential element of the procedural memories inherent in our model. Furthermore, the microglomerular synaptic complexes of multimodal input neurons and Kenyon cell dendrites in the mushroom body calyces might provide “the context in which multimodal integration is performed at discrete loci within the mushroom body lobes” [45], p. 286) and thus might functionally correspond to our motivation network. Heisenberg [47] lists several examples that indeed show that mushroom bodies are required for the stabilization of, and the switching between, different modes of behaviour. Experience-related changes in the microglomerular synaptic complex, as they occur in bees [48], [49] as well as *Cataglyphis* ants [50], [51], could indicate that the Kenyon cells at their calycal sites are involved in memory formation. Moreover, the mushroom bodies have long been regarded as centers of multimodal interconnections, especially as regards to the context-dependent processing of multimodal information (e.g., [52], [53]) and decision making (e.g., [54]). Finally, the outputs of local mushroom body circuits reach, via protocerebral neuropils, the central complex [46], which is responsible for the higher-order control of oriented walking [55]–[57].

## Supporting Information

Figure S1 Searching paths. When a simulated zero-vector ant starts a searching movement, the distance to the starting position shows a temporal development similar to that observed in real ants (compare with [12], Figure 8). Distance d (length of current vector) vs. time.
(TIF)

Figure S2 The density profile of searching paths (mean values from n = 10 simulated searching paths) is in good agreement with profiles recorded from real ants performing search paths (compare with [12], Figure 5).
(TIF)

Text S1 Computational details. Further information is given concerning the structure of the motivation network, the simulation of landmark networks, and a brief discussion comparing the application of Cartesian or polar coordinate systems.
(DOC)

Text S2 Results concerning area concentrated search. Figure 5 shows an individual example of an Area Concentrated Search path. Here we illustrate how the distance between starting position and actual position develops over time (Figure S1) and show the density profile averaged over 10 searching paths to allow for a comparison with biological data.
(DOC)
