# Supplementary figures and images for: No Need for a Cognitive Map: Decentralized Memory for Insect Navigation
Source: PLoS Comput Biol. 2011 Mar 17;7(3):e1002009. doi: 10.1371/journal.pcbi.1002009 (PMC3060166; doi:10.1371/journal.pcbi.1002009)

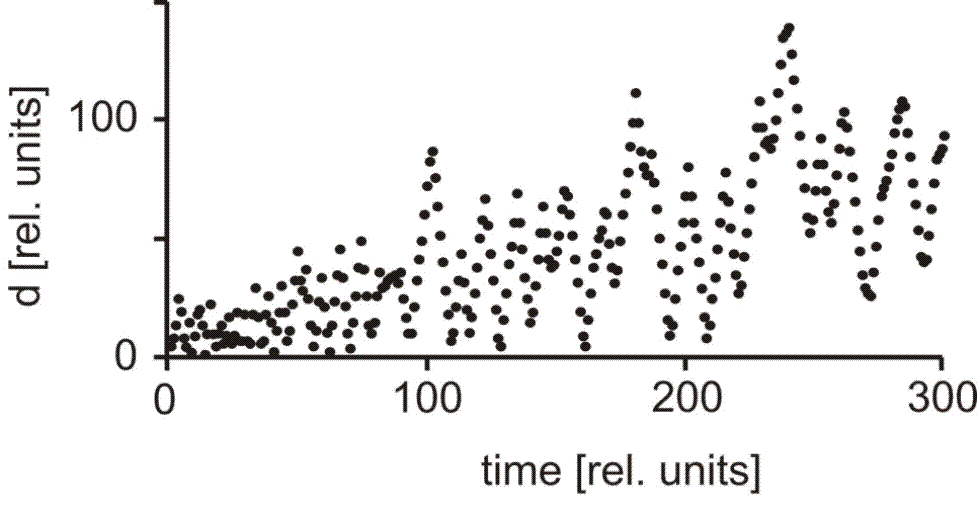

Supplement: Figure S1 — Searching paths. When a simulated zero-vector ant starts a searching movement, the distance to the starting position shows a temporal development similar to that observed in real ants (compare with [12], Figure 8). Distance d (length of current vector) vs. time. (TIF) [file pcbi.1002009.s001.tif]

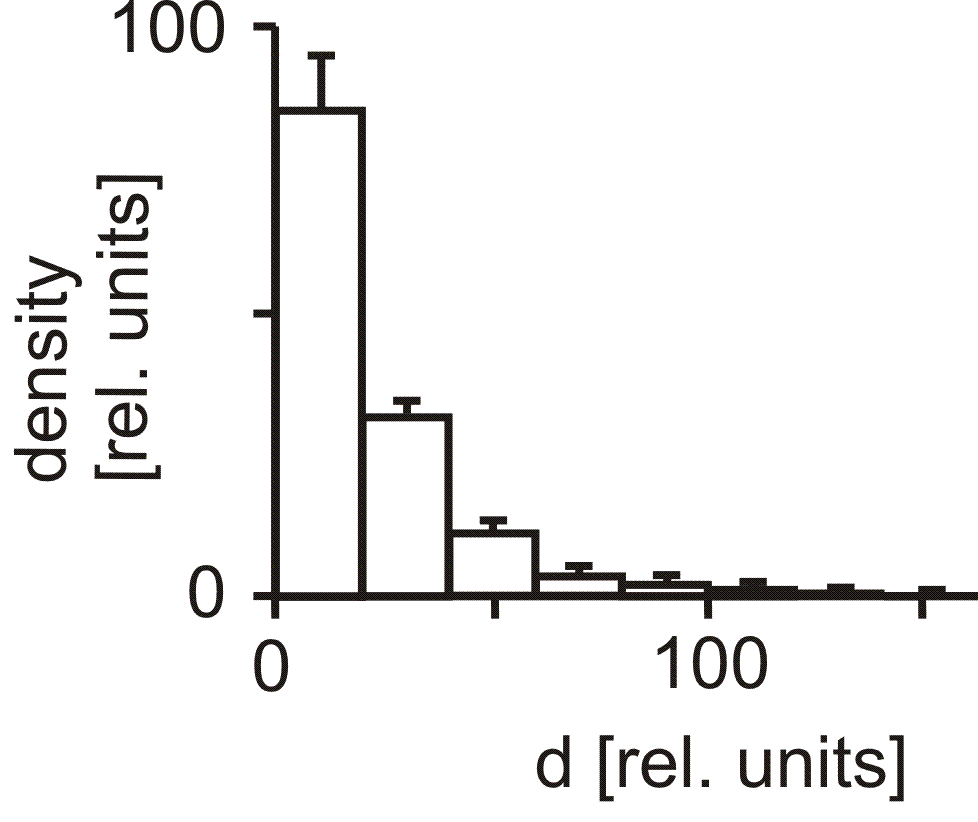

Supplement: Figure S2 — The density profile of searching paths (mean values from n = 10 simulated searching paths) is in good agreement with profiles recorded from real ants performing search paths (compare with [12], Figure 5). (TIF) [file pcbi.1002009.s002.tif]
