# Supplementary material for: No Need for a Cognitive Map: Decentralized Memory for Insect Navigation
Source: PLoS Comput Biol. 2011 Mar 17;7(3):e1002009. doi: 10.1371/journal.pcbi.1002009 (PMC3060166; doi:10.1371/journal.pcbi.1002009)
Supplement: Text S1 — Computational details. Further information is given concerning the structure of the motivation network, the simulation of landmark networks, and a brief discussion comparing the application of Cartesian or polar coordinate systems. (DOC) [file pcbi.1002009.s003.doc]

Text S1

**Computational details**

The *motivation network* is realized by recurrently connected input compensation (IC) units, type suppression units [35,36]. This type of artificial neuron is equipped with a rectifier (negative input values provide zero output) and may be equipped with further nonlinear characteristics. In the actual simulation, in addition to the rectifier, we use an upper bound (output ≤ 1). If no external input is given to any IC unit, the net behaves like a traditional recurrent network. If an external input is given, the input suppresses the activation of this unit which would have resulted from feedback provided by the other units. In other words, the output of this unit is fully controlled by its external input. The main advantage of IC units is given by the fact that there is a simple learning algorithm available to train recurrent neural networks consisting of IC units. This is however not yet exploited in the actual simulation.

*Simulation of landmark*s: Recurrent neural networks (RNN) consisting of IC units could have also been used to realize procedural memories (for the home landmark net see [34]). Other solutions are given by [37-39] which are computationally simpler, but require a large memory space to store the snapshot. The proposal used by Cruse and Hübner [34] requires only very small memory, but is, in principle, computationally more complex as it required the capability to recognize objects (e.g. figure – ground separation) and to determine the vector pointing from the agent to the object. As these capabilities are here assumed to be given, the simulation is straightforward. Application of networks consisting of IC units to represent the procedure using route landmarks was also possible, but would have required a slightly more complex RNN in order to represent the forgetting term. To keep the simulation as simple as possible, we implemented the procedural memories in a traditional, algorithmic way.

*Coordinate systems:* A further technical detail should be mentioned here. Angle values used in the memory elements (main text, Fig. 1, dashed arrows) are internally represented by Cartesian (x,y) rather than polar (α,r) coordinates. Of course, the latter would have been possible, but calculation of summation of angle values is much easier when using Cartesian coordinates. Vector length is computed from Cartesian coordinates within each memory element to determine the salience value. Note that Cartesian coordinate values are only used locally and are not exchanged between memories. In other words, we deal with a distributed memory and not with the global coordinate system of a cognitive map. Interestingly, a detailed study by Vickerstaff and Cheung [30] shows that application of geocentric Cartesian coordinates is advantageous compared to other solutions. Recently, these authors [40] have presented even stronger arguments for the application of an allocentric/geocentric Cartesian-like representation in path integration system that has to deal with noise.
