# Supplementary material for: No Need for a Cognitive Map: Decentralized Memory for Insect Navigation
Source: PLoS Comput Biol. 2011 Mar 17;7(3):e1002009. doi: 10.1371/journal.pcbi.1002009 (PMC3060166; doi:10.1371/journal.pcbi.1002009)
Supplement: Text S2 — Results concerning area concentrated search. Figure 5 shows an individual example of an Area Concentrated Search path. Here we illustrate how the distance between starting position and actual position develops over time (Figure S1) and show the density profile averaged over 10 searching paths to allow for a comparison with biological data. (DOC) [file pcbi.1002009.s004.doc]

Text S2

**Results Concerning Area Concentrated Search**

As explained in the Methods section, we have implemented a procedural routine that generates an ‘area concentrated search’ comparable to the one observed in real ants [12]. A simulation example is given in Fig. 5 of the main text. A more detailed evaluation of the simulations showed that average distance to the imaginary position of the home site increases with searching time and, as illustrated in Fig. S1, the agent, like ants, returns to that home position in irregular periods (compare with [12], Fig. 8). Furthermore, there is a search density profile which decreases with the length of the current vector, roughly approximating a power function (Fig. S2). Comparison with data from Wehner and Srinivasan ([12], their Fig. 5) shows that there is qualitative agreement. It might be worth emphasizing that in our model no switch is required to trigger homing. Only a clock measuring search duration has to be started at the beginning of the search.
